# Adaptive responses and transgenerational plasticity of a submerged plant to benthivorous fish disturbance

**DOI:** 10.1002/ece3.10398

**Published:** 2023-07-31

**Authors:** Fuchao Li, Zhenjun Zuo, Haocun Zhao, Weicheng Yu, Haihao Yu, Dan Yu, Chunhua Liu

**Affiliations:** ^1^ The National Field Station of Freshwater Ecosystem of Liangzi Lake, Department of Ecology, College of Life Sciences Wuhan University Wuhan China

**Keywords:** asexual propagules, benthivorous fish, stoichiometric homeostasis, submerged plants, transgenerational plasticity

## Abstract

Submerged macrophytes play a key role in the restoration of shallow eutrophic lakes. However, in some subtropical lakes, benthivorous fishes dominate the fish assemblages and influence the growth of submerged plants. A comprehensive understanding of the direct and indirect effects of benthivorous fishes on submerged plants is important. We conducted mesocosm experiments to examine the effects of three densities of benthivorous fish, *Misgurnus anguillicaudatus*, on the water properties, the growth, asexual reproduction, and the germination of turions of *Potamogeton crispus* L. Our results showed that fish disturbance increased TN, TP, PO_4_–P, NH_4_–N, and NO_3_–N of the water, raising the extinction coefficient K, Chl *a*, and the periphyton biomass. Benthivorous fish disturbance reduced the total biomass, root length, relative growth rate (RGR), and branching number while increasing the plant height of *P. crispus*. The P stoichiometric homeostasis coefficient (*H*_P_) (except turions) and *H*_N_ was lower in plant tissues due to fish disturbance. Benthivorous fish disturbances promoted turions formation (e.g., increased turions total numbers and biomass) of *P. crispus*. Moreover, *P. crispus* exhibited transgenerational plasticity for benthivorous fish affecting turion emergence. The maximum final germination rate occurred only when fish density in the mother plant grow experiment matched that in the turion germination experiment. Turions generated by *P. crispus* disturbed by low‐density fish exhibited increased germination rates. Our findings suggest that controlling benthivorous fish reduces its indirect and direct effects on submerged vegetation, facilitating the successful restoration of these plants.

## Graphical abstract

The disturbance caused by benthivorous fish can have long‐lasting detrimental effects on water quality. Not only does it reduce the growth rate of submerged plants, but it also diminishes their capacity for P‐enrichment. Furthermore, the asexual reproduction of plants and germination of offspring are impacted. By improving the management of benthivorous fish, we can aid in the recovery of vegetation.

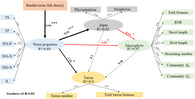

## 1 INTRODUCTION

Increased nutrient availability (N and P loading) in shallow lakes typically results in high biomass of algae and decreased water transparency, resulting in loss of submerged macrophytes (Sayer et al., 2010), causing severe damage to lake ecosystem functions (Capers, 2003). Restoration of eutrophic shallow lakes relies on reestablishing submerged vegetation (Jeppesen et al., 1997). However, single restoration measures, such as reducing exogenous nutrient loads or planting submerged plants alone, do not lead to plant recovery (Hilt et al., 2006). High fish activity levels (herbivory, disturbance, etc.) may jeopardize restoration efforts (Chen, Su, et al., 2020; Dorenbosch & Bakker, 2012; Gu et al., 2018). Recently, the fast recovery of fish biomass and dominance by benthivorous fish in subtropical and tropical lakes have been widely observed (Clavero et al., 2023; Roshni et al., 2022). Therefore, it is essential to reveal the effects of benthivorous fish disturbance on submerged plants.

Benthivorous fish have a high potential for causing sediment resuspension, which harms water characteristics (Badiou & Goldsborough, 2015; Chen, Liu, et al., 2020). However, some previous studies have provided conflicting evidence, such as PO_4_‐P increase (Chen, Liu, et al., 2020; Chen, Su, et al., 2020; He et al., 2022), decrease (Badiou & Goldsborough, 2015), or no change (Gu et al., 2018). Overlying water Chl *a* increase (Badiou & Goldsborough, 2015; Ren et al., 2022) or no change (Chen, Su, et al., 2020). Those may be attributed to their studies' experimental periodicity and benthivorous fish density. Through excretion and disturbance, benthivorous fishes increase the nutrient content of overlying water, promote the growth of phytoplankton and periphyton, and form a shaded effect on submerged plants (Alirangues Nuñez et al., 2023; Ren et al., 2022). Therefore, benthivorous fish disturbance hurts the growth and reestablishment of aquatic plants in shallow lakes (Pacheco et al., 2021; Ren et al., 2022).

Plants retain the relative stability of their stoichiometric features in the face of fluctuations in the availability of elements in the external environment, a process known as stoichiometric homeostasis (Cooper, 2008; Elser et al., 2000). Plant ecological stoichiometry instability reflects plants' ability to adapt to changing environments (Sterner & Elser, 2002), and is influenced by nutrient availability (Rao et al., 2021), light intensity, fertilization (Sterner & Elser, 2002), plant organs, growth stages and elements (Li et al., 2018). Active plant tissue organs such as leaves exhibit higher stoichiometric homeostasis (Zhang et al., 2018), with the P stoichiometric homeostasis coefficient (*H*_P_) positively correlated with submerged plants biomass (Rao et al., 2021). High *H*_P_ plants possess higher RGR and can enrich more P (Li et al., 2018), aiding P removal in eutrophic lakes. Increased P levels also promote submerged plant development by increasing reproductive inputs (Qian et al., 2014; Yan et al., 2021). Fish disturbance can release sediment nutrients into the water column, potentially increasing plant reproduction. However, Chen, Liu, et al. (2020) and Chen, Su, et al. (2020) found that plant reproduction, measured by the number of branches and divisions, was reduced, suggesting that low‐light stress outweighs increased nutrient benefits. Plants may respond by increasing investment in nutritional organs at the expense of reproduction. The involvement of N and P elements in biochemical synthesis is closely related to resource allocation (Sterner & Elser, 2002). Increased nutrient content due to fish disturbance may result in N and P buildup in plant organs, affecting *H*_N_ and *H*_P_ in tissues and organs and altering coordination between reproductive and trophic organs. Few studies have examined fish disturbance effects on submerged plant stoichiometric homeostasis.

The environment of a parent plant can significantly influence offspring adaptability (Dong et al., 2017) through means such as modifying plant morphology (Yamauchi et al., 2021) or altering seed or vegetative propagule nutrient quantity and quality (Dong et al., 2017). Maternal effects are often considered adaptive in sexually reproducing plants, particularly when ancestors can predict the environments of their offspring (Herman & Sultan, 2011). Recent research suggests that animal‐induced maternal effects may also play a significant role in asexually reproducing species (Dong et al., 2017; Yamauchi et al., 2021), though these studies mainly focus on the herbivorous level. Regulation measures for fish populations can indirectly affect submerged plant growth environments by altering benthivorous fish abundance. This unstable environment can influence a plant mother's assessment of her offspring's growth environment, potentially impacting germination. However, little is known about this.

*Potamogeton crispus* L is a widely distributed submerged plant that typically produces summer‐dormant turions (Wu et al., 2009). Turions are the dominant ways of propagation for local recruitment in *P. crispus* persistence and range expansion (Yan et al., 2021). It is also frequently chosen as a pioneer species for lake restoration due to its strong growth rates, quick multiplication, ability to resist low light, and excellent removal with organics and nitrogen (Wu et al., 2009; Yan et al., 2021). *Misgurnus anguillicaudatus*, belonging to the family *Cobitidae*, is a common species of small benthivorous fish found in Chinese lakes and has a strong ability to induce sediment resuspension (Canal et al., 2015). In this experiment, we study the effects of three densities of *M. anguillicaudatus* on water properties, submerged plant growth, asexual reproduction, and propagule germination of *P. crispus*, and we hypothesized that (1) the disturbance of benthivorous fish increases the nutrient content of water and promotes the growth of algae, which is not conducive to submerged plants; (2) the high‐nutrient and low‐light environment caused by fish disturbance will increase P and N in all tissues and organs of *P. crispus*; (3) under the long‐term influence of fish disturbance, *P. crispus* allocate more resources to reproductive organs and improve the adaptability of offspring to the maternal environment, such as increasing the germination rate of turions.

## 2 MATERIALS AND METHODS

### 2.1 Experimental material

This experiment was conducted at The National Field Station of the Freshwater Ecosystem of Liangzi Lake, Hubei Province, China (30°05′–30°18′ N, 114°21′–114°39′ E). The seedlings of *P. crispus* of similar size were collected from Liangzi Lake on 20 January 2021. The initial height of *P. crispus* was 19.12 ± 0.95 cm (mean ± SD), and the initial dry mass was 0.027 ± 0.004 g (mean ± SD). The shoots were carefully washed to remove the attachments before being planted in tanks. A total of 12 outdoor experimental tanks (100 cm in diameter and 100 cm in height) were used as our experimental containers. The clay sediment (0.5 mg g^−1^ total phosphorus [dry weight, DW], 3.7 mg g^−1^ total nitrogen [DW], and 2.3% [DW] total organic content) was collected from Liangzi Lake. A 10 cm layer of lake sediment and 90 cm of lake water (1.03 mg L^−1^ for total nitrogen and 0.026 mg L^−1^ for total phosphorus) were added to each tank. To ensure the homogeneity of the sediment conditions, the sediment was thoroughly mixed before the experiment. Similarly, before the start of the experiment, the water quality was tested to ensure that the beginning values were consistent.

*M.anguillicaudatus*, a common benthivorous fish widely found in the mid‐lower Yangtze lakes, was bought from a market near Liangzi Lake. The fish were of similar sizes (10.30 ± 0.14 cm in length and 5.06 ± 0.2 g wet weight) and were acclimatized in lake water for three days before the experiment.

### 2.2 Experimental design

A single factorial design experiment was conducted with three densities of fish (FD), that is, 0, 1, and 4 *M. anguillicaudatus* (0, 7, and 28 g m^−2^), respectively (Figure S1). The densities were chosen based on observations during field surveys in lakes in the middle and lower reaches of the Yangtze River (Gu et al., 2018). At the beginning of the experiment, 192 shoots of plants were planted in 12 tanks; each tank contained 16 shoots, which were chosen based on observations during field surveys in Liangzi Lake (Qian et al., 2014). *M. anguillicaudatus* was added to the corresponding tanks 4 weeks after planting the shoots. To accurately measure the potential periphyton biomass on the submerged plants, a polyethylene (PE) board was utilized. The board, measuring 8 × 5 cm, was suspended vertically at a depth of 50 cm in each mesocosm (Gu et al., 2018). Each tank was covered with thin gauze (5‐mm mesh size) to prevent the fish from jumping out of the tank. During the experiment, the water temperature remained between 7.1°C and 31.2°C. Lake water was added daily to maintain a constant water level of 100 cm. The experiment ended when most of the brown and hardened turion developed during the late growth period of *P. crispus*. Each treatment included four replicates. The experiment lasted from 1 February to 21 May 2021.

At the end of the experiment, mature turions (0.332 ± 0.1 g FW) were collected randomly from three fish density groups for the germination experiment. After collection, the turions were washed crudely with tap water to remove algae and then stored at 4°C in darkness until the experiments began in October 2021 to break the dormancy of the turions (Hay et al., 2008).

The germination experiment aimed to see if the maternal environment affected the germination percentage (Figure S1). Seven hundred twenty turions randomly collected from three fish density treatment groups (FD) were planted in 12 tanks, and each tank contained 60 turions, which included 20 turions from each FD group. Each turion was labeled separately according to the FD treatments in the first experiment, placed in the same clay sediment 2 cm deep, and filled with lake water. The treatment fish density for the germination experiment was defined as three fish densities (0, 7, and 28 g m^−2^), referred to as MD. Four replications were made in this germination experiment. During the experiment, the water temperature remained between 7.6°C and 20.2°C. Lake water was added daily to maintain a constant water level of 100 cm. The experiment was continued until no more turions germinated over 15 consecutive days. The germination experiment lasted from 15 October to 15 December 2021.

### 2.3 Sampling and measurements

Water samples were meticulously collected weekly from each mesocosm throughout the first experiment. An acrylic tube sampler was used to extract 1 L samples. The water quality characteristics were closely monitored, with a total of 15 measurements taken. The samples were analyzed for nutrient contents and chlorophyll *a* (Chl *a*). As a surrogate for phytoplankton biomass, Chl *a* was measured using a handheld probe (HYDROLAB DS5; HACH). Total phosphorus (TP) was analyzed by the Total Phosphorus Analyzer (IL500P; HACH). The colorimetric method was used to measure TN by following digestion with K_2_S_2_O_8_ and NaOH solution. The colorimetric method using Nessler's reagent was used to determine ammonia nitrogen (NH_4_–N), while the UV spectrophotometric method was used to assess nitrate nitrogen (NO_3_–N) (Moss et al., 1996). PO_4_–P was determined by the molybdenum blue method (Gaudet, 1971). Photosynthetic active radiation (PAR) was measured at the water depth of 0 and 0.5 m using an underwater radiation sensor (UWQ‐192S) connected to a data logger (Li‐1400; Li‐Cor Company). Light extinction coefficient (*K*) in the water was calculated as *K* = (ln*I*_1_–ln*I*_2_)/(*d*_2_–*d*_1_), where *d* is water depth, and subscript stands for water depth order: *d*_1_ is the water surface, and *d*_2_ is the deeper position. *I*_1_ and *I*_2_ are PAR at water depths *d*_1_ and *d*_2_, respectively (Duarte et al., 1986).

The leaf chlorophyll concentration was assessed from 0.5 g fresh and ripe leaves plucked from the petiole on the final sampling day; the samples were put in 10 mL of 80% acetone in the dark for 48 h at 25°C and then quantified by colorimetric analysis using a spectrophotometer (Yan et al., 2021). The PE board was carefully removed by hand from each mesocosm and placed in a ziplock plastic bag to analyze periphyton biomass in the laboratory. The periphyton (a mixture of algae, bacteria, and residue, but primarily algae) attached to the board was rinsed with distilled water and filtered through precombusted and preweighed GF/C filters, and subsequently dried in the oven at 70°C for 48 h. All plants were harvested and carefully washed with tap water for later measurements. The branching number of *P. crispus* in each tank was counted. Five individuals with shoot lengths were chosen for the size of plant height. The plant height was considered the maximum shoot length for *P. crispus*. All the plants were divided into leaf, root, stem, and turion. To determine the dry weight (DW), all leaves, roots, and stems were dried with bibulous paper, weighed, oven‐dried at 70°C to constant weight, and weighed. Twenty turions of each tank were randomly selected to measure the length and width, oven‐dried at 70°C to constant weight, and weighed to determine the dry weight (DW). The remaining biomass was calculated according to the relative proportion. In each tank, the total biomass of each species was the sum of the DW of each plant organ. The following formula calculated the RGR of the plants in each tank: RGR = ln (*M*_2_/*M*_1_)/days, where *M*_2_ and *M*_1_ were the plant dry weight at the end and beginning of the experiment, respectively, and days were the duration of the experiment.

The dried plant and sediment samples were meticulously ground into a fine powder to determine C, N, and P. An elemental analyzer (UNICUBE; Elementar) was then used to analyze the N and C content in both plant tissues and sediment. The P content of the macrophyte and sediment was determined using a sulfuric acid‐hydrogen peroxide digestion and ammonium molybdate–antimony potassium tartrate–ascorbic acid spectrophotometric method (Richard & Donald, 1996). As submerged macrophytes absorb nutrients from sediment and water column (Carignan & Kalff, 1980), the sediment element contents (P) and water column element contents (N) were utilized as the external nutrient supply. This allowed for the calculation of the stoichiometric homeostasis coefficient (*H*) of plants. The coefficient can be accurately determined using the following equation (Sterner & Elser, 2002): log(*y*) = log(*c*) + (1/*H*) log(*x*), where *y* is the element content (N or P) of the plant tissues, *x* is the element content (N and P) of external nutrient resources, and *c* is a constant. The community *H*_N_ and *H*_P_ are the weighted sums of the tissues and organs.

During the germination experiment, the same water physical and chemical indexes as the mother experiment were detected every 2 weeks, and a total of 4 monitoring sessions were conducted. After the germination experiment, the final germination rate of turions is counted for all turions after harvest.

### 2.4 Statistical analyses

To test the effects of three fish densities on water quality, we used repeated measures analysis of variance (ANOVA) to analyze time series data. One‐way analysis of variance (ANOVA) was used to test the statistical significance of the differences in the growth and physiological indices. Two‐way ANOVA was used to test the effects of macrophyte organ, treatment, and their interactions on stoichiometric coefficients. The effects of FD and MD on the final germination rate were analyzed using generalized linear models (GLMs), decomposing R^2^ afterward with the “glmm.hp” R package (Lai et al., 2022). The least significant difference (LSD) was used for multiple comparisons. Before analysis, using box‐cox conversions as needed. A schemer‐Ray‐Hare nonparametric test was used to test data that do not conform to normal distributions and the Kruskal–Wallis test for multiple comparisons, such as TC, TN, and C: N of macrophyte. Statistical significance was assigned at *p* < .05. All data analyses were carried out using SPSS 23.0 (SPSS) and R (v3.60), and statistical graphics were drawn in Origin 2021 (Origin Lab Corp.).

To quantify the relationship between benthivorous fish density, water characters, and macrophyte growth, we fitted partial least squares path models (PLS‐PM) using data at the end of the experiment. Partial least squares path models (PLS‐PM) were carried out in the “pls‐pm” package in R (v3.60).

## 3 RESULTS

### 3.1 Water properties

**FIGURE 1:**
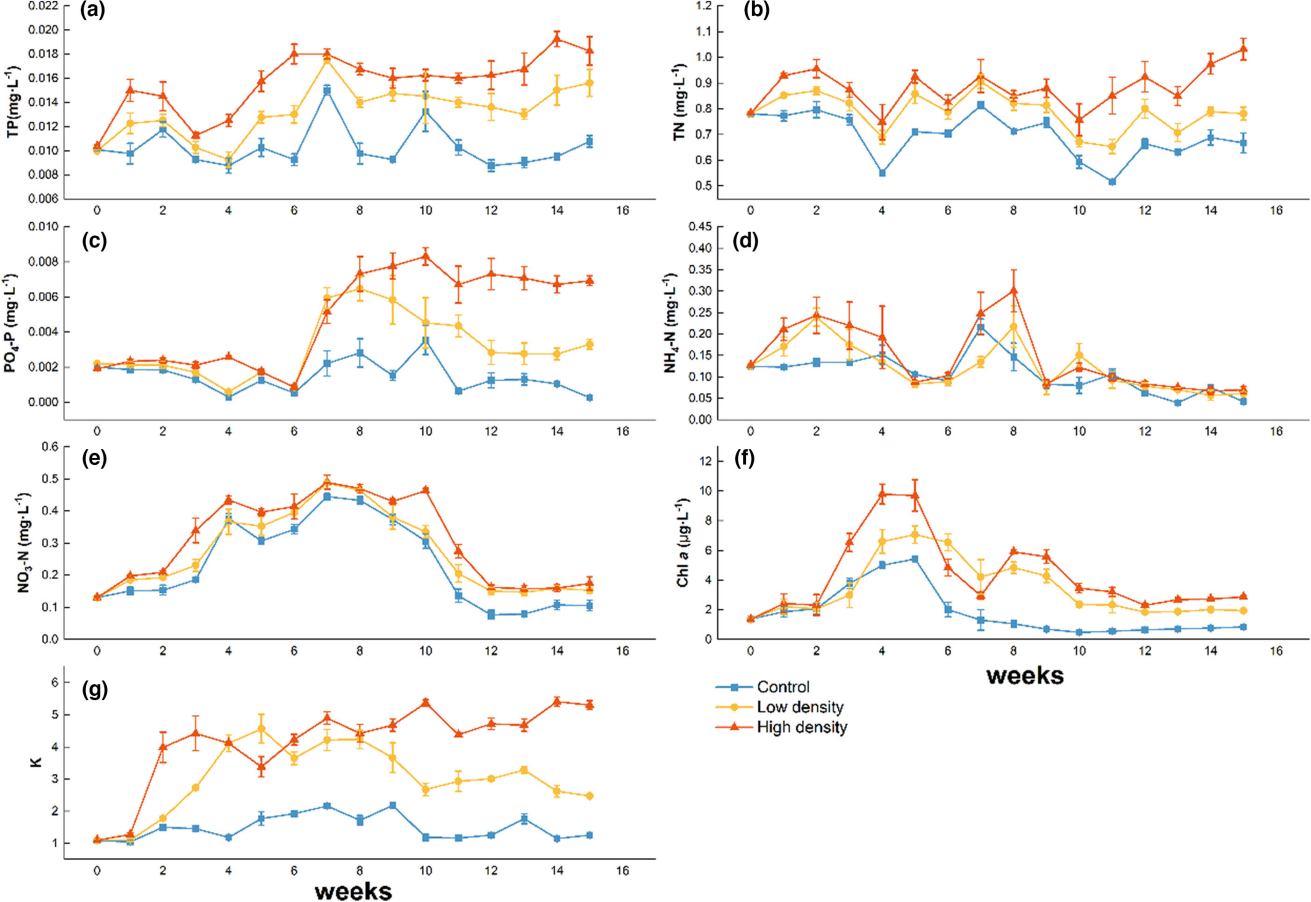
(a) Total nitrogen (TN), (b) total phosphorus (TP), (c) orthophosphate (PO_4_–P), (d) nitrate nitrogen (NO_3_–N), (e) ammonia (NH_4_–N), (f) light extinction coefficient (K) and (g) Chl *a* in different treatments during the experiment. Values are means of the four replicates; error bars represent standard deviation.

There was a significant effect of fish density on the TN, TP, NO_3_–N, NH_4_–N, PO_4_‐P, Chl *a*, and K, and the results changed with time (*p* < .05) (Figure 1, Table 1). During the experiment, the mean TP in the low‐density and high‐density fish groups was 32% and 58% greater than the control group (Figure 1a). Mean TN in the low‐density and high‐density fish groups were 15% and 30% greater than the control groups, respectively (Figure 1b). Mean PO_4_–P in the low‐density and high‐density fish groups were 195% and 440% greater than the control group (Figure 1c). Mean NO_3_–N in the low‐density and high‐density fish groups were 30% and 49% greater than the control groups, respectively (Figure 1d). Mean NH_4_–N in the low‐density and high‐density fish groups were 21% and 39% greater than the control groups, respectively (Figure 1e). In water clarity, the mean *K* in the low‐density and high‐density fish groups was 109.9% and 201.3% greater than those of the control group throughout the experiment period (Figure 1f). Mean Chl *a* in the low‐density and high‐density fish groups were 183% and 257% greater than those of the control group throughout the experiment period (Figure 1g).

**TABLE 1:** Results of repeated measures analysis of variance (ANOVA) for water chemical parameters and water clarity based on time‐weighted data during the experiment.

	df	TP		TN		PO_4_–P		NO_3_–N	
		*F*	*p*	*F*	*p*	*F*	*p*	*F*	*p*
FD	2	68.53	**<.001**	117.4	**<.001**	148.5	**<.001**	77.66	**<.001**
Time	15	18.92	**<.001**	17.174	**.008**	22.432	**<.001**	131.46	**<.001**
FD × Time	30	4.12	**<.001**	2.722	**<.001**	7.626	**<.001**	3.553	**<.001**
		**NH**_**4**_ **–N**		**K**		**Chl *a***			
FD	2	6.684	**.017**	598.3	**<.001**	107.7	**<.001**		
Time	15	32.79	**<.001**	93.82	**<.001**	39.276	**<.001**		
FD × Time	30	2.31	**<.001**	18.89	**<.001**	7.465	**<.001**		

*Note*: Significant effects were marked in bold.

Abbreviation: FD, fish density.

### 3.2 Macrophyte morphophysiological traits

**FIGURE 2:**
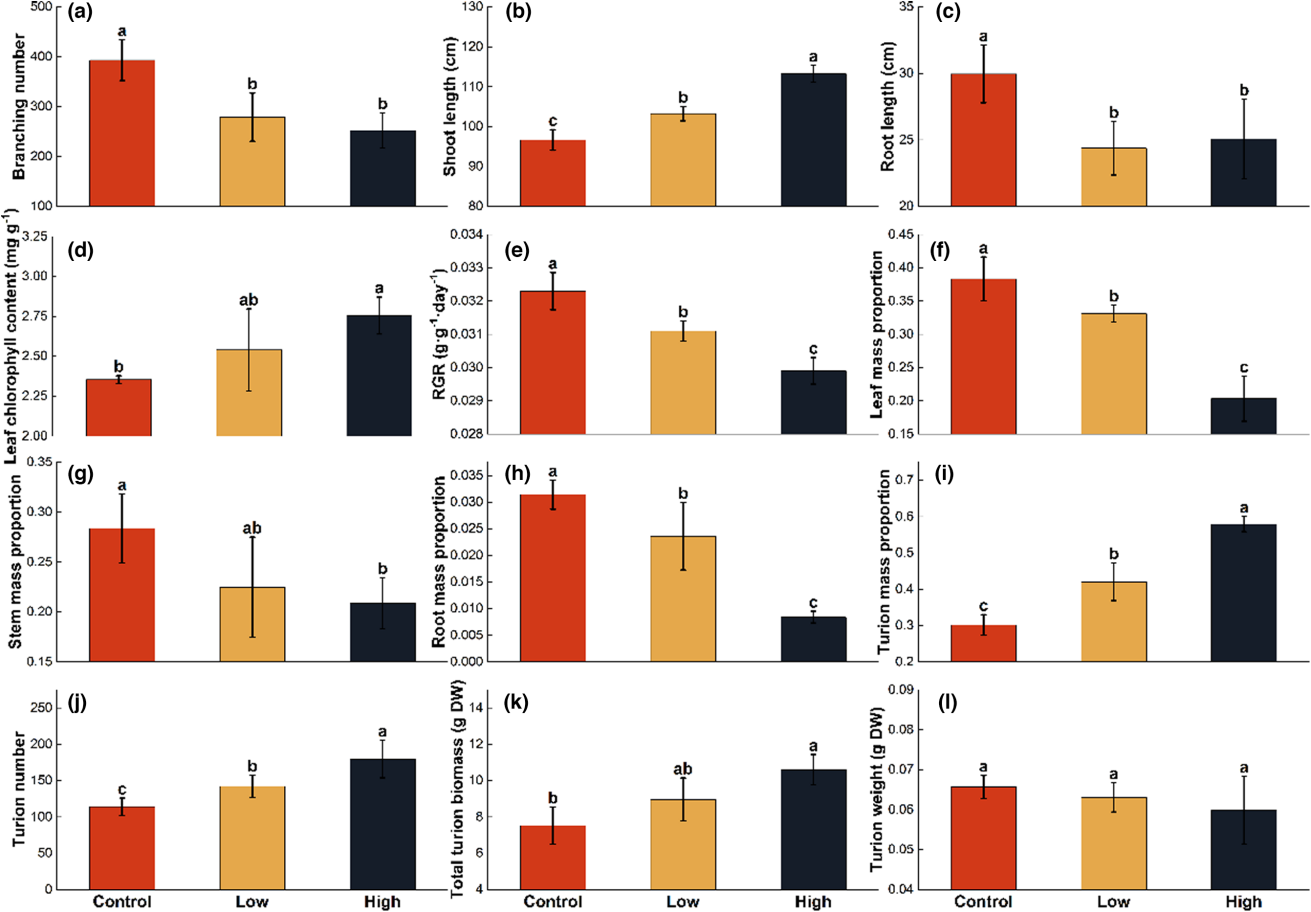
Morphophysiological traits of *P. crispus* in different fish density treatments. (a) Branching number, (b) shoot length, (c) root length, (d) leaf chlorophyll content, (e) relative growth rate (RGR), (f) leaf mass proportion, (g) stem mass proportion, (h) root mass proportion, (i) turion mass proportion, (j) turion number, (k) total turion biomass, (l) turion weight. Values represent mean ± SE. The bars with different letters above are significantly different (*p* < .05).

Except for the turion weight, fish density significantly affected all morphophysiological traits (Table 2). Both fish density groups significantly decreased branching number, root length, and RGR (Figure 2a,c,e) while increasing shoot length and the chlorophyll content of plant leaves (Figure 2b,d). Regarding the biomass allocation of *P. crispus*, with the increase in fish density, the biomass proportion of leaf and root decreased significantly (Figure 2f,g,h). However, plants grown at fish treatments show an apparent increase in the turion fraction (Figure 2i).

**TABLE 2:** One‐way ANOVA for morphophysiological traits of macrophytes.

	Fish density		
	df	*F*	*p*
Branching number	2	12.788	**.002**
Shoot length(cm)	2	58.174	**<.001**
Root length(cm)	2	6.321	**.019**
Leaf chlorophyll content (mg g^−1^)	2	6.114	**.021**
RGR (g g^−1^ day^−1^)	2	31.519	**<.001**
Leaf mass proportion	2	42.902	**<.001**
Stem mass proportion	2	4.261	**.05**
Root mass proportion	2	33.645	**<.001**
Turion mass proportion	2	59.492	**<.001**
Turion number	2	14.023	**.002**
Total turion biomass (g DW)	2	9.108	**.007**
Turion weight (g DW)	2	1.089	.377

*Note*: Significant effects were marked in bold.

In the control group, 15% of the turions were still immature at the harvest, and the turions in the group with fish were all mature. The appearance of the turions in the high‐density fish group was 1 week earlier than in the control group. Both low‐ and high‐density fish treatments significantly increased the turion number by 24.6% and 57.7% and turion biomass by 19.3% and 41% (Figure 2j,k), respectively. However, fish treatments did not affect the individual turion weight (Figure 2l).

### 3.3 Stoichiometric characteristics

**FIGURE 3:**
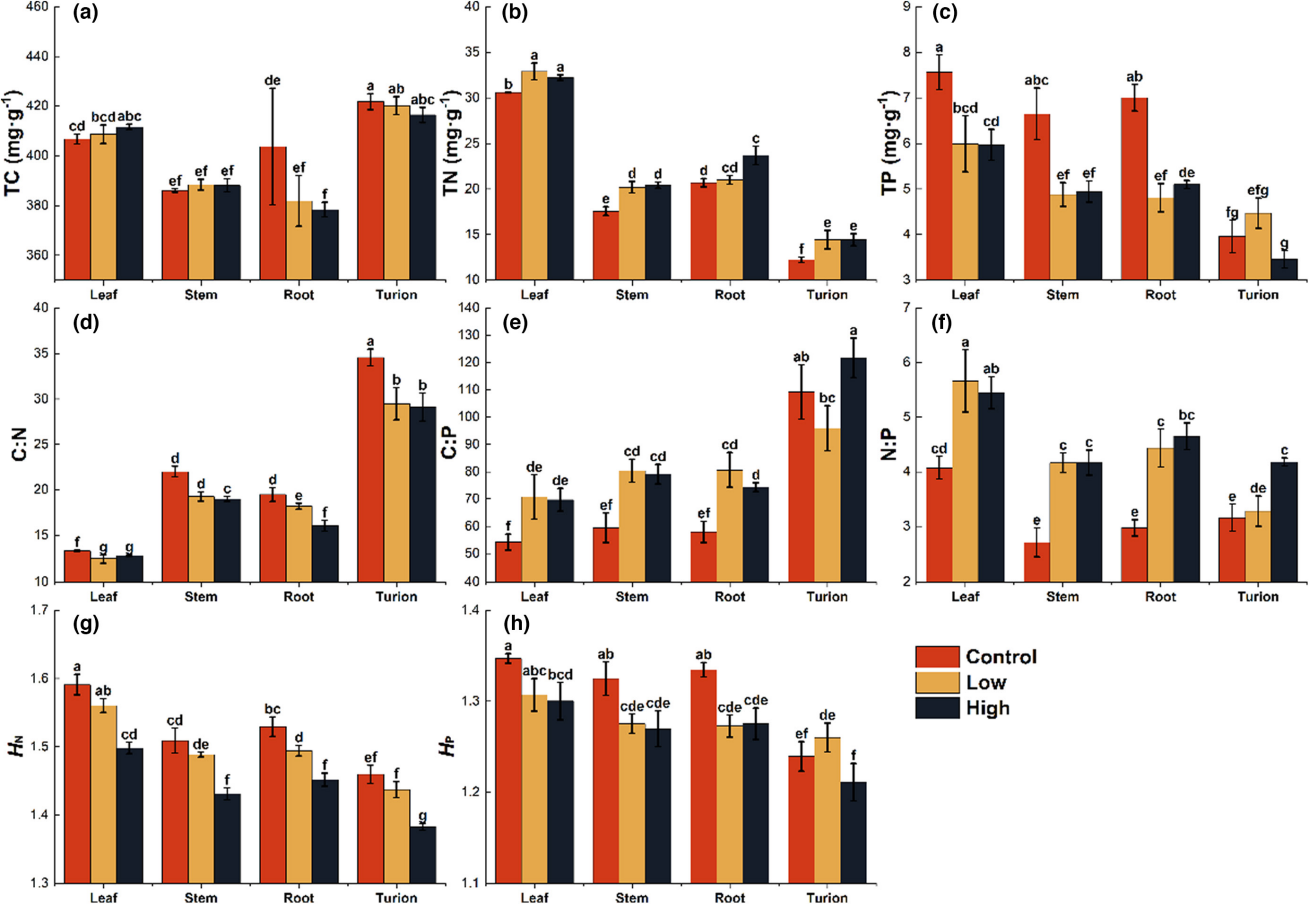
The stoichiometric characteristics of different organs of *P. crispus*. (a) TC, (b) TN, (c) TP, (d) C:N, (e) C:P, (f) N:P, (g) *H*_N_ (homeostasis coefficient of N), (h) *H*_P_ (homeostasis coefficient of P). Values represent mean ± SE. The bars with different letters above are significantly different (*p* < .05).

Except for the TC of root under high fish density treatment, *P. crispus* was less affected by fish treatments (Figure 3a). Both fish treatments significantly increased the TN and N:P while decreasing the C: N of *P. crispus* (Figure 3b,d,e). Fish treatments significantly reduced the TP of the leaf, stem, and root, while the C:P of the three organs showed the opposite pattern (Figure 3c,e). Fish density treatment had no significant effects on turions' TP and C:P (Figure 3c,e). Compared with the control group, only high‐density fish disturbance decreased the *H*_N_ in all organs (Figure 3g). Both fish density groups led to significantly lower *H*_P_ of stem and root, while only high fish density treatment significantly decreased *H*_P_ of leaf and turion (Figure 3h). The high‐density group had the lowest *H*_P_ (Figure S3a).

### 3.4 Turions germination characters

**FIGURE 4:**
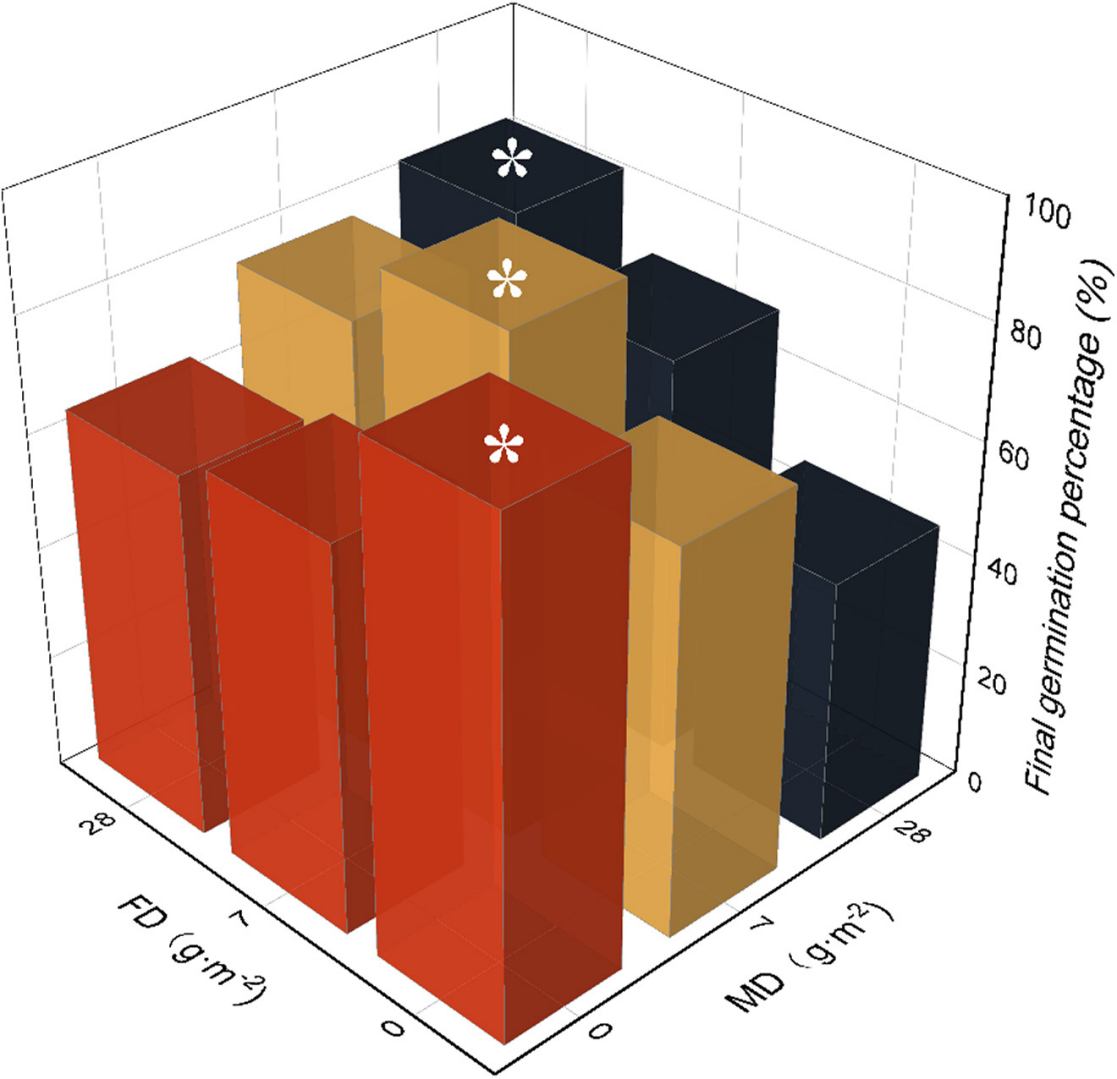
The final germination percentage of the in situ germination experiment. Different colors present different fish density (FD) groups. Different colors represent different MD groups. The asterisk indicates the highest germination percentage of each FD group (*p* < .05).

FD and MD dramatically affected the final germination percentage (Table S1). In the same MD, there was a significant difference in final germination percentage under different FDs. Turions exhibited maximum germination percentage only when MD was consistent with FD (Figure 4). For instance, turions under MD_0_ treatment had only the highest germination percentage (87.5%) in the FD_0_ condition. Similarly, turions under MD_7_ treatment exhibited only the highest germination percentage (87.5%) in the FD_7_ condition. In other words, the most suitable germination environment was the same as its growing environment. The lowest germination (46.25%) was found under treatment FD_0_× MD_28_.

The interaction of FD and MD explained 46.89% of the germination results, and FD and MD alone explained 9.53% and 43.58% (Figure S4).

### 3.5 Relationship between *M. anguillicaudatus* density, water properties, and *P. crispus*

**FIGURE 5:**
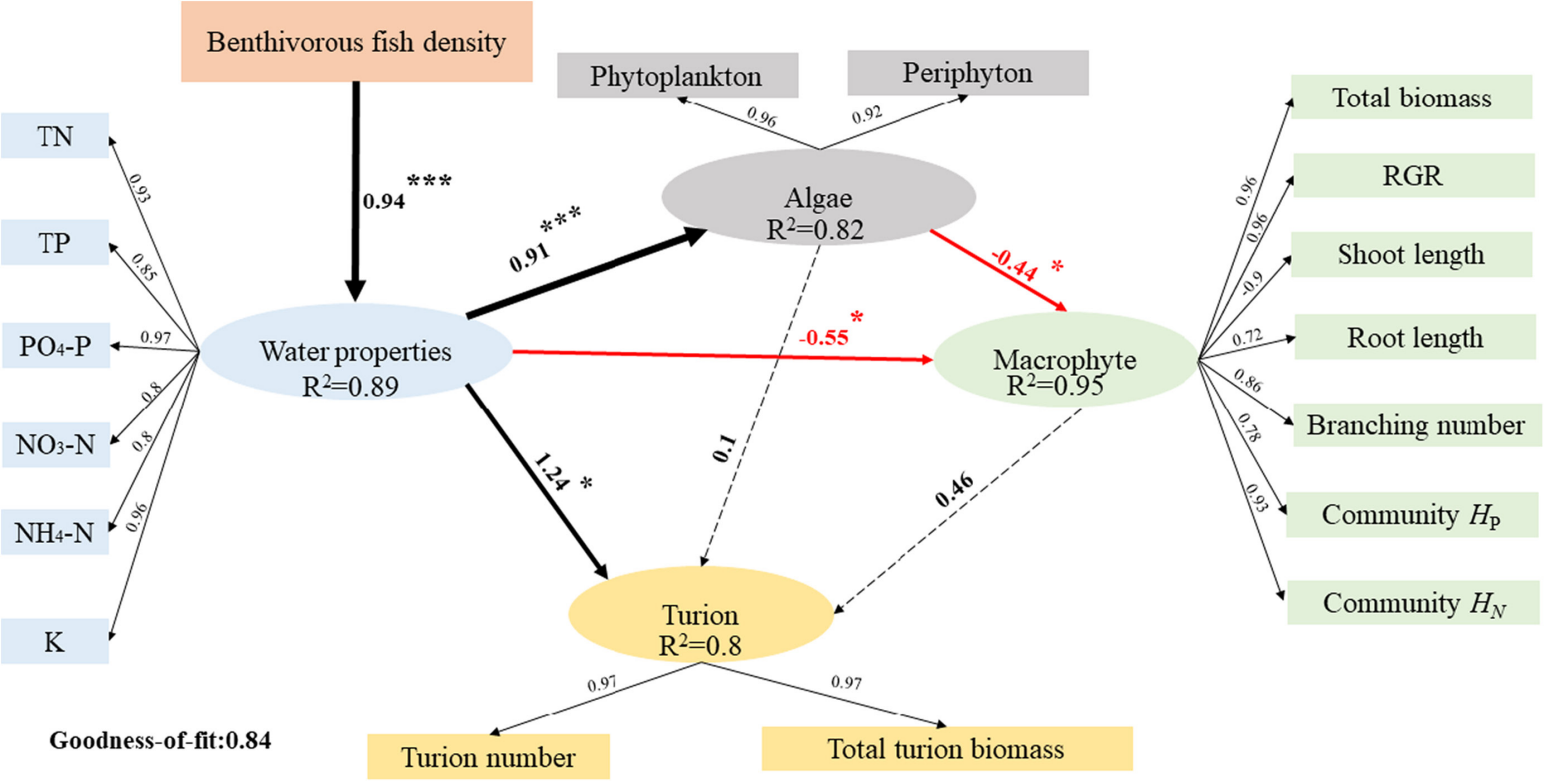
Partial least squares path models based on the final sampling data depict benthivorous fish density's direct and indirect effects on macrophyte growth and reproduction. Red, black, and dashed arrows represent negative, positive, and nonsignificant paths. The thickness of the significant paths represents the magnitude of the standardized regression coefficient or effect sizes, given on the arrows. *R* ^2^s for component models are given on the endogenous variables. Significant paths are marked with a star. (Significant *p* values: *p < .05, **p < .01, ***p < .001).

PLS‐PM revealed direct and indirect relationships between benthivorous fish density, water properties, macrophytes, and turion indicators (Figure 5). All water body characteristics and algal indicators were significantly and positively correlated with fish density and were included in the analysis. Plant and turion indicators were selected according to the degree of effect and scientific experience. For macrophyte traits, the shoot length was positively correlated with water properties and algae. In contrast, the total biomass, RGR, branching number, root length, community *H*_P_ and community *H*_N_ were negatively correlated with water properties and algae (Figure 5). Turion indicators are positively associated with water properties (Figure 5). In conclusion, water properties were critical direct drivers of macrophyte and turion indicators, and fish disturbance indirectly affects *P. crispus* growth and reproduction through water properties (Figure 5).

## 4 DISCUSSION

### 4.1 Effects of benthivorous fish activity on water properties

Our experimental results revealed significant differences in the water column physicochemical properties for all fish treatments compared to previous short‐term studies. Notably, even the experimental high‐density fish treatment was only 28 g m^−2^, significantly lower than the 128 g m^−2^ observed in previous studies (Ren et al., 2022). As hypothesized, benthivorous fish disturbance increased water column TP and TN levels. High‐nutrient levels significantly increased phytoplankton and periphyton biomass (Ren et al., 2022) and the Chl *a* of water as seen in their study. However, Chen, Liu, et al. (2020) and Chen, Su, et al. (2020) observed no effect on Chl *a* in their study, potentially due to lower benthivorous fish density and shorter experimental period. Fish disturbance also increased water particulate matter, with high planktonic algae density increasing water K and reducing light availability for plants (Ren et al., 2022). The significant PO_4_–P content increase in the fish treatment group may result from benthivorous fish disturbance releasing nutrients from the substrate to overlying water. Conversely, low‐light environments caused by shading hurt *P. crispus*. Reduced nutrient utilization efficiency and metabolism (Rhew et al., 1999) resulted in chlorosis and brittle leaves easily broken by fish disturbance. Withered and broken leaves or shoots would sink and release nutrients into the water column as they decayed (Cao et al., 2004).

### 4.2 Growth, reproductive strategies, and intergenerational plasticity of *P. crispus*

Fish disturbance reduced the total biomass and the RGR of *P. crispus* with fewer branches and lower root length (Figure 2a,c,e). Benthivorous fish disturbance increased K and caused light limitation for plants, while higher periphyton and phytoplankton density (Figure 5) competed with submerged plants for both light and nutrient resources (Chen, Liu, et al., 2020; Ren et al., 2022). *P. crispus* increase the chlorophyll content of leaves and the shoot length (Figure 2b, d), aiding adaptation to low‐light stress (Chen, Liu, et al., 2020; Ferreira et al., 2016). However, it was difficult to fully compensate for low light's adverse effects on submerged plants (He et al., 2017), resulting in the reduced growth of *P. crispus*. Chen, Liu, et al. (2020) and Chen, Su, et al. (2020) also found fewer submerged plant branches due to low light caused by fish disturbance. Additionally, fish disturbance disrupted sediment structure (Canal et al., 2015), negatively impacting *P. crispus* root growth and nutrient uptake capacity (Eissenstat et al., 2015), further reducing growth.

Turions of *P. crispus* are vegetative, dormant organs that form in response to unfavorable ecological conditions (Adamec, 2018). Our results confirm our hypothesis that fish disturbance increased the total biomass and turion numbers (Figure 2j,k). This may result from several mechanisms. High TN and TP in water caused by fish disturbance may have contributed to the formation of turions, consistent with previous studies finding strong P‐enrichment in water increased the number of turions of *P. crispus* (Qian et al., 2014). Higher plant leaf chlorophyll content (Figure 2d) under fish disturbance could increase the net photosynthetic rate, stimulating turion formation (Adamec, 2018). Escape strategies often rely on reproductive success before the onset of severe stress (Campos et al., 2004), increasing resources for reproduction to ensure offspring continuation (Garcia & Eubanks, 2019), or accelerating production (Lucas‐Barbosa et al., 2013). Only turions had developed in this experiment, with no flowers or fruits yet emerging. The fish disturbance caused earlier and greater turion maturation, increasing turion amount and total biomass at the expense of stem, leaf, and root biomass. Turions serve as propagation and dispersal means with greater longevity (Jian et al., 2003). We interpret our results as a strategy for offsprings to escape from stressful habitats of maternal plants. Moreover, *P. crispus* turions can act as storage organs buffering against unfavorable ecological conditions (Jian et al., 2003), with plants in stressed habitats allocating more dry mass to storage organ development than plants in favorable habitats (Puijalon et al., 2008). Our study suggests that fish disturbance may increase periphyton and phytoplankton biomass, leading to competition with *P. crispus* for light and nutrients, thereby contributing to the early and more abundant formation of turions.

Transgenerational effects generally enhance offspring performance in response to stressful and benign conditions (Yin et al., 2019). Our study revealed that the disturbance caused by benthivorous fish significantly impacted the final germination rate of turions. Interestingly, we found that the maximum germination rate was only achieved under maternal conditions. For germination experiments, we selected mature turions, observing that turion traits such as weight, TC, and TP (Figures 2i and 3a,c) remained unchanged across three fish density treatments, suggesting developmental time had little influence on turion traits. Notably, turions are a product of *P. crispus* asexual reproduction, with unchanged DNA sequences (Latzel & Klimešová, 2010). We found only a weak correlation between the final germination rate under each treatment and turion traits and water environment (Figure S5), suggesting that germination rate differences may result from epigenetic inheritance. Further molecular research is needed to explore this mechanism in greater detail. Turions affected by low‐density fish disturbance showed greater germination ability in this experiment (Figure 4). Human activities such as the 10‐year fishery ban in the middle and lower reaches of the Yangtze River or fish regulation measures in eutrophic lake restoration activities may change benthivorous fish density in shallow lakes, potentially impacting aquatic plant propagule bank germination.

### 4.3 Influence on stoichiometric characteristics

High‐nutrient and low‐light environments affect plant photosynthesis, influencing photosynthates and total carbon (TC) content. However, plants have compensatory mechanisms, such as resource allocation among organs and tissues (Rao et al., 2020). Fish disturbance reduced root TC considerably, while leaf, stem, and turion TC remained unchanged (Figure 3a). This may be due to reduced carbohydrate production in leaves and decreased carbon transport to roots caused by fish disturbance‐induced changes in the nutrient and light environment (Rao et al., 2020). Plants maintain germination by delivering more nutrients to turions formed by leaf specialization (Marschner & Marschner, 2012) and investing more carbon in stem elongation for light capture in response to low light (Marschner & Marschner, 2012; Schneider et al., 2014). Benthivorous fish disturbance increased total nitrogen (TN) in all organs, possibly due to increased water body nitrogen content. Submerged plants can absorb nutrients via shoots and roots, leading to excessive element uptake (Sterner & Elser, 2002) and increased plant nitrogen content (Rao et al., 2020). Low light caused by fish disturbance is closely related to plant photosynthetic rate (Marschner & Marschner, 2012), so *P. crispus* increases TN in leaves and stems to adapt. Fish disturbance reduced total phosphorus (TP) in leaves, stems, and roots while increasing N:P and C:P ratios in all tissues and organs (Figure 3c,e,f), supporting the growth rate hypothesis (Sterner & Elser, 2002). However, this contradicts our hypothesis that the responses of the N and P in organs are not parallel. Plants primarily take up nitrogen from the water column and phosphorus from sediment (Carignan & Kalff, 1980), but fish disturbance releases sediment nutrients into the water column. However, sediment P content remained unchanged between treatment groups at the end of the experiment (Figure S3d). This may be due to reduced root biomass and length caused by low‐light environment weakening plant root phosphorus uptake and utilization, offsetting sediment P release by fish disturbance. Benthivorous fish disturbance weakens submerged plant P‐enrichment and uptake capacity, hindering shallow‐water eutrophic lake restoration.

## 5 CONCLUSION

Our study shows that benthivorous fish *M. anguillicaudatus* not only had a long‐term adverse effect on water properties but also affected submerged plant growth and P‐enrichment capacity. *P. crispus* increased the number of turion through the early formation of turion to adapt to fish disturbances, and the benthivorous fish effect can persist across vegetative generations. Additionally, turion germination rates of mother plants formed by fish disturbance were higher. Early control of benthivorous fish during water restoration is beneficial for submerged plant growth and vegetation formation.

## AUTHOR CONTRIBUTIONS

**Fuchao Li:** Conceptualization (lead); data curation (lead); formal analysis (equal); investigation (lead); methodology (lead); software (lead); visualization (lead); writing – original draft (lead); writing – review and editing (lead). **Zhenjun Zuo:** Conceptualization (equal); data curation (equal); formal analysis (equal); investigation (equal); software (equal); writing – original draft (equal). **Haocun Zhao:** Conceptualization (equal); formal analysis (equal); investigation (equal); methodology (equal); writing – original draft (equal). **Yu Weicheng:** Conceptualization (equal); formal analysis (equal); investigation (equal); methodology (equal); writing – original draft (equal). **Yu Haihao:** Conceptualization (equal); formal analysis (equal); investigation (equal); methodology (equal); writing – original draft (equal). **Dan Yu:** Conceptualization (equal); formal analysis (equal); project administration (lead); resources (equal); supervision (equal); validation (equal). **Chunhua Liu:** Conceptualization (equal); funding acquisition (equal); project administration (equal); supervision (lead); validation (lead); writing – original draft (equal); writing – review and editing (lead).

## FUNDING INFORMATION

This research was financially supported by the Fundamental Research Funds for the Central Universities (2042020kf1025).

## CONFLICT OF INTEREST STATEMENT

The authors declare that they have no known competing financial interests or personal relationships that could have appeared to influence the work reported in this paper.

## Supporting information


Appendix S1

## Data Availability

All data used in the production of this article are available via Dryad: https://doi.org/10.5061/dryad.msbcc2g3q.

## References

[ece310398-bib-0001] Adamec, L. (2018). Ecophysiological characteristics of turions of aquatic plants: A review. Aquatic Botany, 148, 64–77. 10.1016/j.aquabot.2018.04.011

[ece310398-bib-0002] Alirangues Nuñez, M. M. , Hussner, A. , Mauersberger, R. , Brämick, U. , Hühn, D. , He, L. , & Hilt, S. (2023). Periphyton and benthivorous fish affect charophyte abundance and indicate hidden nutrient loading in oligo‐ and mesotrophic temperate hardwater lakes. Freshwater Biology, 68, 312–324. 10.1111/fwb.14026

[ece310398-bib-0003] Badiou, P. H. J. , & Goldsborough, L. G. (2015). Ecological impacts of an exotic benthivorous fish, the common carp (*Cyprinus carpio* L.), on water quality, sedimentation, and submerged macrophyte biomass in wetland mesocosms. Hydrobiologia, 755, 107–121. 10.1007/s10750-015-2220-6

[ece310398-bib-0004] Campos, H. , Cooper, M. , Habben, J. E. , Edmeades, G. O. , & Schussler, J. R. (2004). Improving drought tolerance in maize: A view from industry. Field Crops Research, 90, 19–34. 10.1016/j.fcr.2004.07.003

[ece310398-bib-0005] Canal, J. , Laffaille, P. , Gilbert, F. , Lauzeral, C. , & Buisson, L. (2015). Influence of temperature on surface sediment disturbance by freshwater fish: A microcosm experiment. Annales de Limnologie‐International Journal of Limnology, 51, 179–188. 10.1051/limn/2015012

[ece310398-bib-0006] Cao, T. , Ni, L. , & Xie, P. (2004). Acute biochemical responses of a submersed macrophyte, *Potamogeton crispus* L., to high ammonium in an aquarium experiment. Journal of Freshwater Ecology, 19, 279–284. 10.1080/02705060.2004.9664542

[ece310398-bib-0007] Capers, R. S. (2003). Six years of submerged plant community dynamics in a freshwater tidal wetland. Freshwater Biology, 48, 1640–1651. 10.1046/j.1365-2427.2003.01115.x

[ece310398-bib-0008] Carignan, R. , & Kalff, J. (1980). Phosphorus sources for aquatic weeds: Water or sediments? Science, 207, 987–989. 10.1126/science.207.4434.987 17830461

[ece310398-bib-0009] Chen, J. , Su, H. , Zhou, G. , Dai, Y. , Hu, J. , Zhao, Y. , Liu, Z. , Cao, T. , Ni, L. , Zhang, M. , & Xie, P. (2020). Effects of benthivorous fish disturbance and snail herbivory on water quality and two submersed macrophytes. Science of the Total Environment, 713, 136734. 10.1016/j.scitotenv.2020.136734 32019051

[ece310398-bib-0010] Chen, J. F. , Liu, Z. G. , Xiao, S. F. S. , Chen, R. Z. , Luo, C. Q. , Zhu, T. S. , Cao, T. , Ni, L. Y. , Xie, P. , Su, H. J. , & Zhang, M. (2020). Effects of benthivorous fish disturbance on chlorophyll a contents in water and the growth of two submersed macrophytes with different growth forms under two light regimes. Science of the Total Environment, 704, 135269. 10.1016/j.scitotenv.2019.135269 31796282

[ece310398-bib-0011] Clavero, M. , Suh, J. , Franch, N. , Aparicio, E. , Buchaca, T. , Caner, J. , Garcia‐Rodriguez, S. , Llimona, F. , Pou‐Rovira, Q. , Rocaspana, R. , & Ventura, M. (2023). Invaders they are a‐changing: A recent, unexpected surge of invasive loaches in Catalonia. Freshwater Biology, 68, 621–631. 10.1111/fwb.14051

[ece310398-bib-0012] Cooper, S. J. (2008). From Claude Bernard to Walter Cannon. Emergence of the concept of homeostasis. Appetite, 51, 419–427. 10.1016/j.appet.2008.06.005 18634840

[ece310398-bib-0013] Dong, B.‐C. , Fu, T. , Luo, F.‐L. , & Yu, F.‐H. (2017). Herbivory‐induced maternal effects on growth and defense traits in the clonal species *Alternanthera philoxeroides* . Science of the Total Environment, 605–606, 114–123. 10.1016/j.scitotenv.2017.06.141 28662425

[ece310398-bib-0014] Dorenbosch, M. , & Bakker, E. S. (2012). Effects of contrasting omnivorous fish on submerged macrophyte biomass in temperate lakes: A mesocosm experiment. Freshwater Biology, 57, 1360–1372. 10.1111/j.1365-2427.2012.02790.x

[ece310398-bib-0015] Duarte, C. M. , Kalff, J. , & Peters, R. H. (1986). Patterns in biomass and cover of aquatic macrophytes in lakes. Canadian Journal of Fisheries and Aquatic Sciences, 43, 1900–1908. 10.1139/f86-235

[ece310398-bib-0016] Eissenstat, D. M. , Kucharski, J. M. , Zadworny, M. , Adams, T. S. , & Koide, R. T. (2015). Linking root traits to nutrient foraging in arbuscular mycorrhizal trees in a temperate forest. New Phytologist, 208, 114–124. 10.1111/nph.13451 25970701

[ece310398-bib-0017] Elser, J. J. , Sterner, R. W. , Gorokhova, E. , Fagan, W. F. , Markow, T. A. , Cotner, J. B. , Harrison, J. F. , Hobbie, S. E. , Odell, G. M. , & Weider, L. W. (2000). Biological stoichiometry from genes to ecosystems. Ecology Letters, 3, 540–550. 10.1111/j.1461-0248.2000.00185.x

[ece310398-bib-0018] Ferreira, V. S. , Pinto, R. F. , & Sant'Anna, C. (2016). Low light intensity and nitrogen starvation modulate the chlorophyll content of Scenedesmus dimorphus. Journal of Applied Microbiology, 120, 661–670. 10.1111/jam.13007 26598940

[ece310398-bib-0019] Garcia, L. C. , & Eubanks, M. D. (2019). Overcompensation for insect herbivory: A review and meta‐analysis of the evidence. Ecology, 100, e02585. 10.1002/ecy.2585 30554427

[ece310398-bib-0020] Gaudet, J. J. (1971). Methods for chemical analysis of fresh waters. H. L. Golterman. The Quarterly Review of Biology, 46, 316–317. 10.1086/406970

[ece310398-bib-0021] Gu, J. , He, H. , Jin, H. , Yu, J. , Jeppesen, E. , Nairn, R. W. , & Li, K. (2018). Synergistic negative effects of small‐sized benthivorous fish and nitrogen loading on the growth of submerged macrophytes – Relevance for shallow lake restoration. Science of the Total Environment, 610–611, 1572–1580. 10.1016/j.scitotenv.2017.06.119 28647154

[ece310398-bib-0022] Hay, F. , Probert, R. , & Dawson, M. (2008). Laboratory germination of seeds from 10 British species of *Potamogeton* . Aquatic Botany, 88, 353–357. 10.1016/j.aquabot.2007.12.010

[ece310398-bib-0023] He, H. , Hu, E. , Yu, J. , Luo, X. , Li, K. , Jeppesen, E. , & Liu, Z. (2017). Does turbidity induced by *Carassius carassius* limit phytoplankton growth? A mesocosm study. Environmental Science and Pollution Research, 24, 5012–5018. 10.1007/s11356-016-8247-z 28000069

[ece310398-bib-0024] He, H. , Qian, T. , Shen, R. , Yu, J. , Li, K. , Liu, Z. , & Jeppesen, E. (2022). Piscivore stocking significantly suppresses small fish but does not facilitate a clear‐water state in subtropical shallow mesocosms: A biomanipulation experiment. Science of the Total Environment, 842, 156967. 10.1016/j.scitotenv.2022.156967 35764152

[ece310398-bib-0025] Herman, J. , & Sultan, S. (2011). Adaptive transgenerational plasticity in plants: Case studies, mechanisms, and implications for natural populations. Frontiers in Plant Science, 2, 102. 10.3389/fpls.2011.00102 22639624 PMC3355592

[ece310398-bib-0026] Hilt, S. , Gross, E. M. , Hupfer, M. , Morscheid, H. , Mählmann, J. , Melzer, A. , Poltz, J. , Sandrock, S. , Scharf, E.‐M. , Schneider, S. , & van de Weyer, K. (2006). Restoration of submerged vegetation in shallow eutrophic lakes – A guideline and state of the art in Germany. Limnologica, 36, 155–171. 10.1016/j.limno.2006.06.001

[ece310398-bib-0027] Jeppesen, E. , Jensen, J. P. , Sondergaard, M. , Lauridsen, T. , Pedersen, L. J. , & Jensen, L. (1997). Top‐down control in freshwater lakes: The role of nutrient state, submerged macrophytes and water depth. Hydrobiologia, 342, 151–164. 10.1023/A:1017046130329

[ece310398-bib-0028] Jian, Y. X. , Li, B. , Wang, J. B. , & Chen, J. K. (2003). Control of turion germination in *Potamogeton crispus* . Aquatic Botany, 75, 59–69. 10.1016/S0304-3770(02)00165-1

[ece310398-bib-0029] Lai, J. , Zou, Y. , Zhang, J. , & Peres‐Neto, P. R. (2022). Generalizing hierarchical and variation partitioning in multiple regression and canonical analyses using the rdacca.hp R package. Methods in Ecology and Evolution, 13, 782–788. 10.1111/2041-210X.13800

[ece310398-bib-0030] Latzel, V. , & Klimešová, J. (2010). Transgenerational plasticity in clonal plants. Evolutionary Ecology, 24, 1537–1543. 10.1007/s10682-010-9385-2

[ece310398-bib-0031] Li, W. , Li, Y. , Zhong, J. , Fu, H. , Tu, J. , & Fan, H. (2018). Submerged macrophytes exhibit different phosphorus stoichiometric homeostasis. Frontiers in Plant Science, 9, 1207. 10.3389/fpls.2018.01207 30158949 PMC6104447

[ece310398-bib-0032] Lucas‐Barbosa, D. , van Loon, J. J. A. , Gols, R. , van Beek, T. A. , & Dicke, M. (2013). Reproductive escape: Annual plant responds to butterfly eggs by accelerating seed production. Functional Ecology, 27, 245–254. 10.1111/1365-2435.12004

[ece310398-bib-0033] Marschner, H. , & Marschner, P. (2012). Marschner's mineral nutrition of higher plants. Academic press.

[ece310398-bib-0034] Moss, B. , Stansfield, J. , Irvine, K. , Perrow, M. , & Phillips, G. (1996). Progressive restoration of a shallow lake: A 12‐year experiment in isolation, sediment removal and biomanipulation. Journal of Applied Ecology, 33, 71–86. 10.2307/2405017

[ece310398-bib-0035] Pacheco, J. P. , Aznarez, C. , Meerhoff, M. , Liu, Y. , Li, W. , Baattrup‐Pedersen, A. , Yu, C. , & Jeppesen, E. (2021). Small‐sized omnivorous fish induce stronger effects on food webs than warming and eutrophication in experimental shallow lakes. Science of the Total Environment, 797, 148998. 10.1016/j.scitotenv.2021.148998 34346382

[ece310398-bib-0036] Puijalon, S. , Bouma, T. J. , Van Groenendael, J. , & Bornette, G. (2008). Clonal plasticity of aquatic plant species submitted to mechanical stress: Escape versus resistance strategy. Annals of Botany, 102, 989–996. 10.1093/aob/mcn190 18854376 PMC2712407

[ece310398-bib-0037] Qian, C. , You, W. H. , Xie, D. , & Yu, D. (2014). Turion morphological responses to water nutrient concentrations and plant density in the submerged macrophyte *Potamogeton crispus* . Scientific Reports, 4, 7079. 10.1038/srep07079 25399866 PMC4233331

[ece310398-bib-0038] Rao, Q. , Su, H. , Deng, X. , Xia, W. , Wang, L. , Cui, W. , Ruan, L. , Chen, J. , & Xie, P. (2020). Carbon, nitrogen, and phosphorus allocation strategy among organs in submerged macrophytes is altered by eutrophication. Frontiers in Plant Science, 11, 524450. 10.3389/fpls.2020.524450 33193470 PMC7604295

[ece310398-bib-0039] Rao, Q. , Su, H. , Ruan, L. , Deng, X. , Wang, L. , Rao, X. , Liu, J. , Xia, W. , Xu, P. , Shen, H. , Chen, J. , & Xie, P. (2021). Stoichiometric and physiological mechanisms that link hub traits of submerged macrophytes with ecosystem structure and functioning. Water Research, 202, 117392. 10.1016/j.watres.2021.117392 34243052

[ece310398-bib-0040] Ren, W. J. , Wen, Z. H. , Cao, Y. , Wang, H. , Yuan, C. B. , Zhang, X. L. , Ni, L. Y. , Xie, P. , Cao, T. , Li, K. Y. , & Jeppesen, E. (2022). Cascading effects of benthic fish impede reinstatement of clear water conditions in lakes: A mesocosm study. Journal of Environmental Management, 301, 113898. 10.1016/j.jenvman.2021.113898 34626943

[ece310398-bib-0041] Rhew, K. , Ochs, R. M. B. A. N. C. A. , & Threlkeld, S. T. (1999). Interaction effects of fish, nutrients, mixing and sediments on autotrophic picoplankton and algal composition. Freshwater Biology, 42, 99–109. 10.1046/j.1365-2427.1999.00464.x

[ece310398-bib-0042] Richard, H. L. , & Donald, L. S. (1996). Methods of soil analysis. Part 3. Chemical methods.

[ece310398-bib-0043] Roshni, K. , Renjithkumar, C. R. , Sreekanth, G. B. , Raghavan, R. , & Ranjeet, K. (2022). Fish community structure and functional guild composition in a large tropical estuary (Vembanad Lake, India). Environmental Science and Pollution Research, 30, 29635–29662. 10.1007/s11356-022-24250-8 36417074

[ece310398-bib-0044] Sayer, C. D. , Burgess, A. M. Y. , Kari, K. , Davidson, T. A. , Peglar, S. , Yang, H. , & Rose, N. (2010). Long‐term dynamics of submerged macrophytes and algae in a small and shallow, eutrophic lake: Implications for the stability of macrophyte‐dominance. Freshwater Biology, 55, 565–583. 10.1111/j.1365-2427.2009.02353.x

[ece310398-bib-0045] Schneider, S. , Pichler, D. , Andersen, T. , & Melzer, A. (2014). Light acclimation in submerged macrophytes: The roles of plant elongation, pigmentation and branch orientation differ among *Chara* species. Aquatic Botany, 120, 121–128. 10.1016/j.aquabot.2014.05.002

[ece310398-bib-0046] Sterner, R. W. , & Elser, J. J. (2002). Ecological stoichiometry: The biology of elements from molecules to the biosphere. Princeton University Press.

[ece310398-bib-0047] Wu, J. , Cheng, S. , Liang, W. , He, F. , & Wu, Z. (2009). Effects of sediment anoxia and light on turion germination and early growth of *Potamogeton crispus* . Hydrobiologia, 628, 111–119. 10.1007/s10750-009-9749-1

[ece310398-bib-0048] Yamauchi, A. , Ikegawa, Y. , Ohgushi, T. , & Namba, T. (2021). Density regulation of co‐occurring herbivores via two indirect effects mediated by biomass and non‐specific induced plant defenses. Theoretical Ecology, 14, 41–55. 10.1007/s12080-020-00479-2

[ece310398-bib-0049] Yan, Z. , Wang, Q. , Li, Y. , Wu, L. , Wang, J. , Xing, B. , Yu, D. , Wang, L. , & Liu, C. (2021). Combined effects of warming and nutrient enrichment on water properties, growth, reproductive strategies and nutrient stoichiometry of *Potamogeton crispus* . Environmental and Experimental Botany, 190, 104572. 10.1016/j.envexpbot.2021.104572

[ece310398-bib-0050] Yin, J. , Zhou, M. , Lin, Z. , Li, Q. Q. , & Zhang, Y.‐Y. (2019). Transgenerational effects benefit offspring across diverse environments: A meta‐analysis in plants and animals. Ecology Letters, 22, 1976–1986. 10.1111/ele.13373 31436014

[ece310398-bib-0051] Zhang, J. , Zhao, N. , Liu, C. , Yang, H. , Li, M. , Yu, G. , Wilcox, K. , Yu, Q. , & He, N. (2018). C:N:P stoichiometry in China's forests: From organs to ecosystems. Functional Ecology, 32, 50–60. 10.1111/1365-2435.12979

