# Correction to ‘Adaptive responses and transgenerational plasticity of a submerged plant to benthivorous fish disturbance’

**DOI:** 10.1002/ece3.10440

**Published:** 2023-08-25

Li, F., Zuo, Z., Zhao, H., Yu, W., Yu, H., Yu, D., & Liu, C. (2023). Adaptive responses and transgenerational plasticity of a submerged plant to benthivorous fish disturbance. Ecology and Evolution, 13, 1–12. https://doi.org/10.1002/ece3.10398.

In the recent article by Li et al. (2023), the citation for ‘glmm.hp’ R package for statistical analysis is incorrect. The correct reference is as follows:

Jiangshan Lai*, Yi Zou, Shuang Zhang, Xiaoguang Zhang. Lingfeng Mao.2022. glmm.hp: an R package for computing individual effect of predictors in generalized linear mixed models. Journal of Plant Ecology 15(6):1302–1307. https://doi.org/10.1093/jpe/rtac096.

We apologize for this error.

