# Supplementary material for: Adaptive responses and transgenerational plasticity of a submerged plant to benthivorous fish disturbance
Source: Ecol Evol. 2023 Jul 31;13(8):10.1002/ece3.10398. doi: 10.1002/ece3.10398 (PMC10390469; doi:10.1002/ece3.10398)
Supplement: Supplementary file 1 — Appendix S1 [file ECE3-13--s001.docx]

## Appendix

**Table S1** The effect of fish density of the maternal experiment (FD) and the density of fish in the germination experiment (MD) on turion germination was studied using a generalized linear model. FD, MD, and their interactions were used as fixed effects, and experimental tank ID was used as a random effect. Bolded values indicate statistical significance at α = 0.05.

|  | Estimate | SE | *t* | *p* |
| --- | --- | --- | --- | --- |
| Intercept | 0.818 | 0.032 | 25.577 | **<0.001** |
| FD | -0.005 | 0.002 | -2.764 | **0.009** |
| MD | -0.01 | 0.002 | -5.305 | **<0.001** |
| FD*MD | 0.001 | 0.001 | 5.328 | **<0.001** |


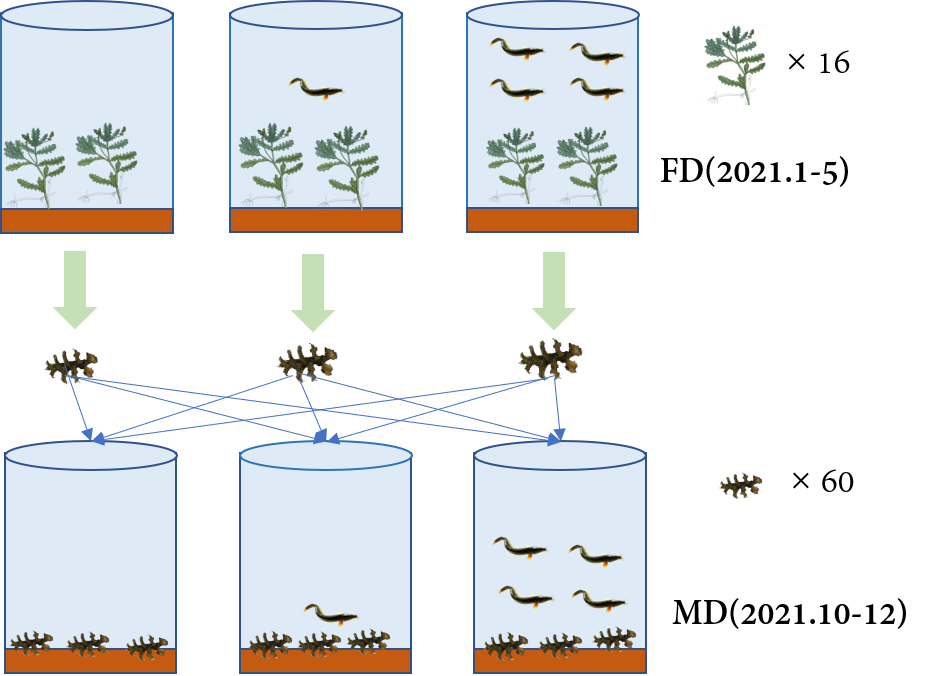


**Figure S1** Schematic diagram of the experimental design.


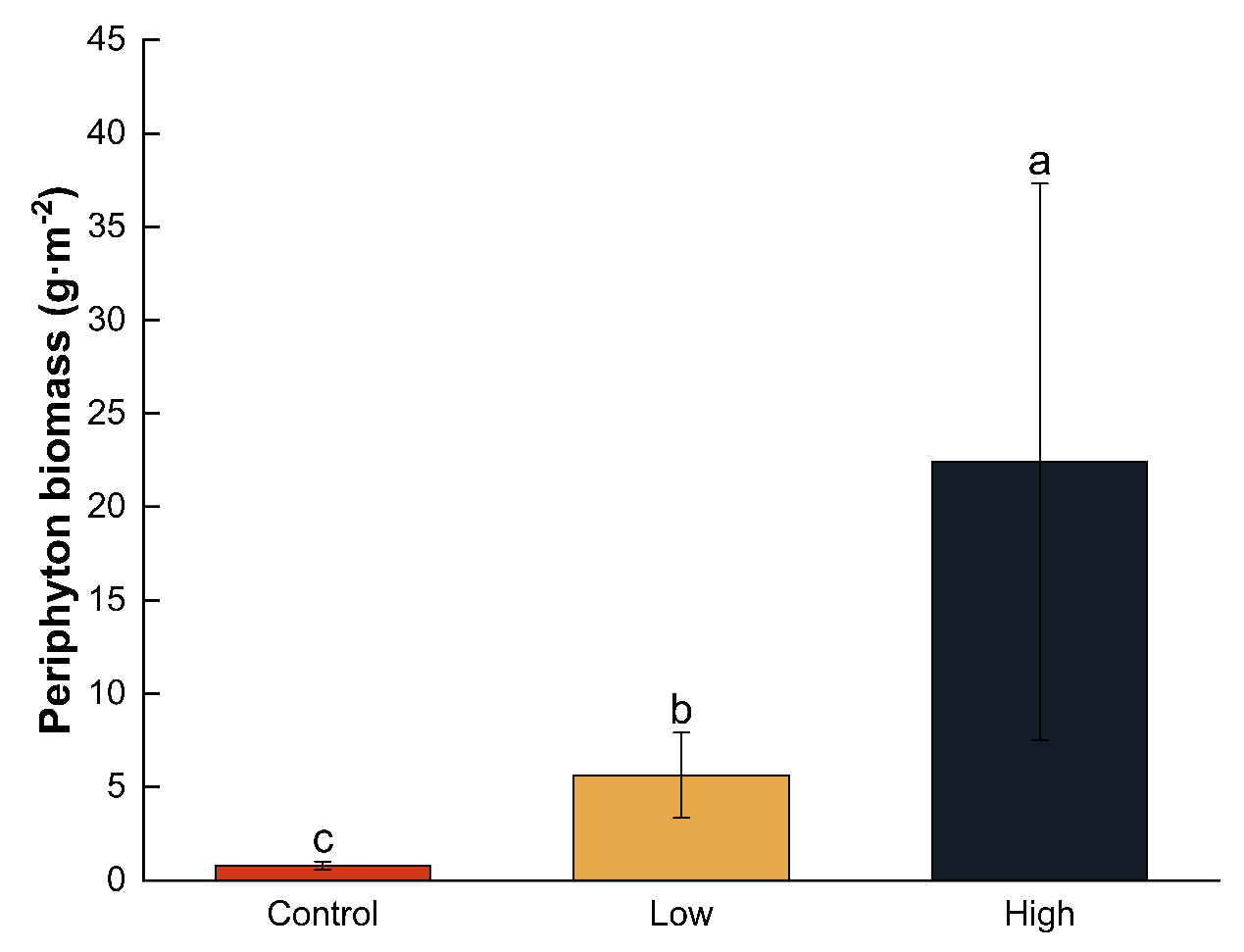


**Figure S2** Biomass of periphyton at the end of the experiment.


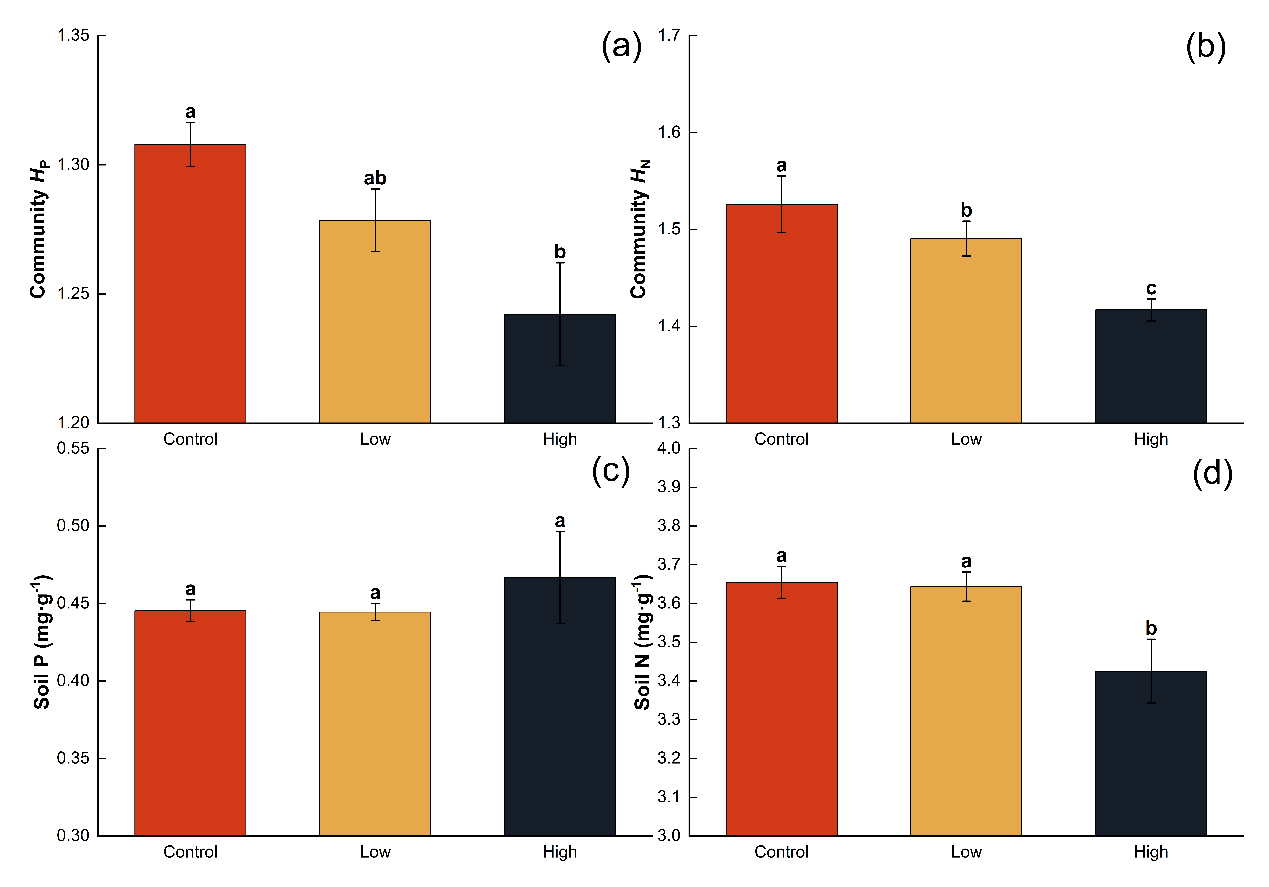


**Figure S3** Community *H*_P_ (a) and community *H*_N_ (b) content as well as sediment N (c), P content (d) at the end of the experiment.


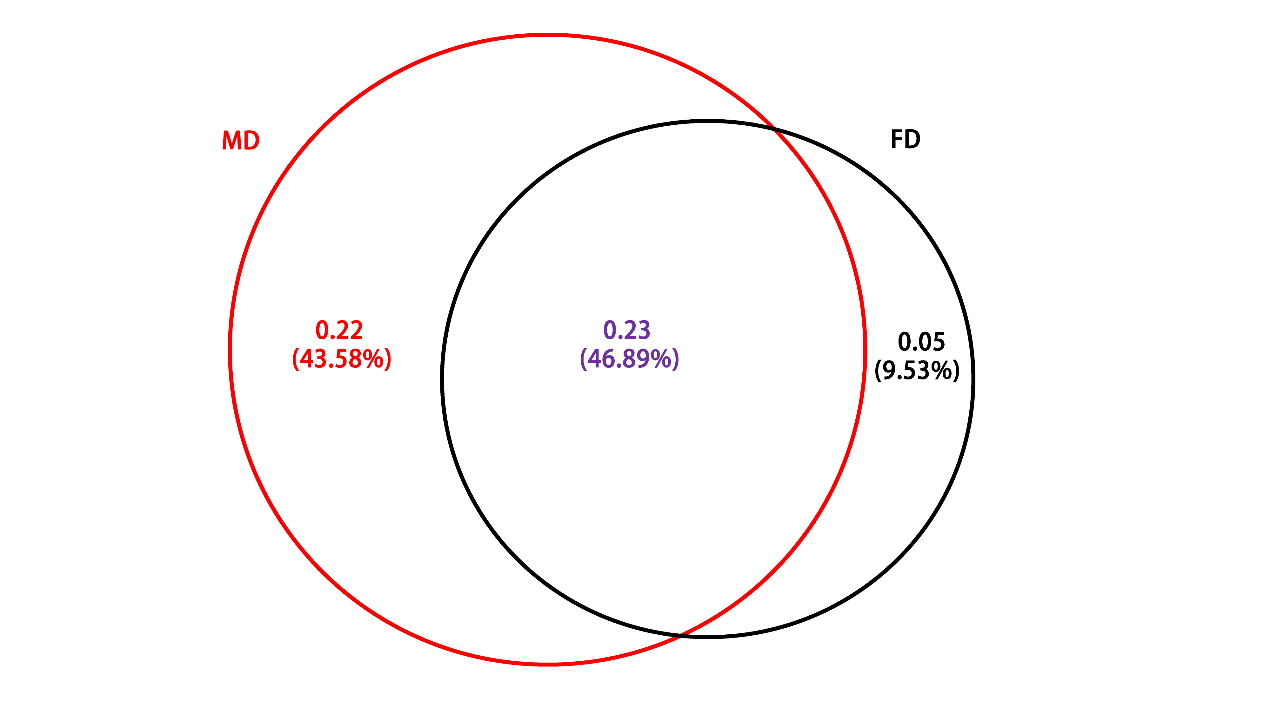


**Figure S4** Explanation rate of the final germination rate of turions by the fish density of maternal experiment (FD) and germination experiment (MD).


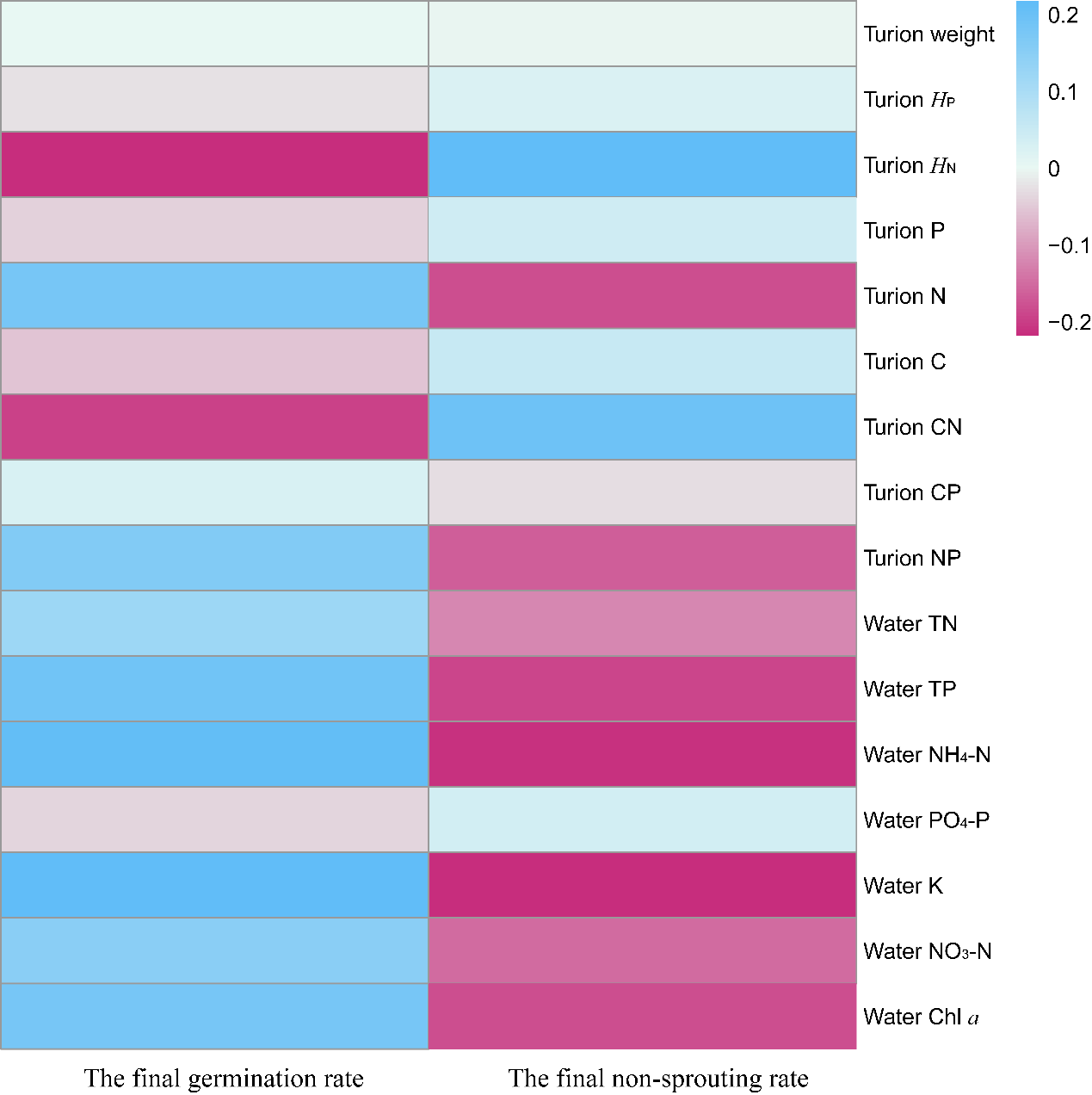


**Figure S5** Correlation between the germination rate of turion under each treatment and turion traits and water column characteristics (mean values during the germination experiment) in germination experiments. (Significant *p* values: **p* < 0.05, ***p* < 0.01, ****p* < 0.001)
